# Development of a single-cell cloning technique for isolation of *Pentatrichomonas hominis*: a promising tool for diagnosing *Trichomonas* spp. infections in the pig breeding industry

**DOI:** 10.1186/s13071-025-06752-9

**Published:** 2025-04-05

**Authors:** Yibin Zhu, Haiming Cai, Shenquan Liao, Juan Li, Siyun Fang, Hanqin Shen, Dingai Wang, Zhuanqiang Yan, Minna Lv, Xuhui Lin, Junjing Hu, Yongle Song, Xiangjie Chen, Lijun Yin, Jianfei Zhang, Nanshan Qi, Mingfei Sun

**Affiliations:** 1 Key Laboratory of Livestock Disease Prevention of Guangdong Province, Key Laboratory of Avian Influenza and Other Major Poultry Diseases Prevention and Control, Ministry of Agriculture and Rural Affairs, Institute of Animal Health, Guangdong Academy of Agricultural Sciences, Guangzhou, 510640 China; 2 Wen’s Group Academy, Wen’s Foodstuffs Group Co., Ltd., Xinxing, 527400 Guangdong China; 3 Guangdong Jingjie Inspection and Testing Co., Ltd., Xinxing, 527400 Guangdong China

**Keywords:** *Trichomonas* spp., Isolation method, Single-cell cloning

## Abstract

**Background:**

Pig breeding is a crucial sector of the global economy, playing a significant role in meat production. However, the prevalence of *Trichomonas* spp., a group of parasites known to induce diarrhea in various hosts, presents significant challenges in breeding facilities. These parasites pose a substantial threat to the pig breeding industry. Furthermore, despite its prevalence, diagnosing *Trichomonas* spp. is often challenging, primarily owing to the presence of mixed infections involving different species within clinical samples. To address this concern, we developed a novel isolation method that combines a single-cell isolation culture technique with an antimicrobial drug susceptibility test.

**Methods:**

Trichomonas was isolated and cultured by using the established single-worm separation technology combined with antibacterial drug screening method, and it was identified as *Pentatrichomonas hominis* by molecular biological identification and morphological identification. The in vitro culture conditions of the isolate were optimized to establish a stable in vitro culture system.

**Results:**

The method developed in this study was effective in successfully isolating a pure species of trichomonad from fecal samples obtained from weaned piglets in Guangdong Province. By optimizing important variables such as the culture medium, serum type, and inoculum quantity, we established a stable in vitro culture system utilizing a modified Diamond medium supplemented with 10% Procell fetal bovine serum without the use of antibiotics. Subsequent analysis of the isolate’s 18S rRNA gene, ITS1-5.8S rRNA-ITS2 gene, and EF-α gene, through polymerase chain reaction, DNA sequencing, and phylogenetic analysis, revealed its close association to *Pentatrichomonas hominis*. Light microscopy and scanning electron microscopy demonstrated the presence of various distinct cellular structures, including four anterior flagella, recurrent flagellum, undulating membrane, pelta and axostyle. Additionally, transmission electron microscopy revealed the existence of organelles such as the Golgi complex, rough endoplasmic reticulum, food vacuoles, and hydrogenosomes. This study represents the first successful isolation of monoclonal cells of *P. hominis* to our knowledge and serves as a valuable baseline for future research focused on the isolation and purification of various other parasites. Additionally, it offers practical guidance for the diagnosis and management of *Trichomonas* spp. infections in pigs.

**Conclusions:**

In summary, our findings underscore the efficacy of our novel isolation technique as a valuable tool for the diagnosis and management of *Trichomonas* spp. infections, which can help mitigate the significant economic losses encountered in the pig breeding industry.

**Graphical Abstract:**

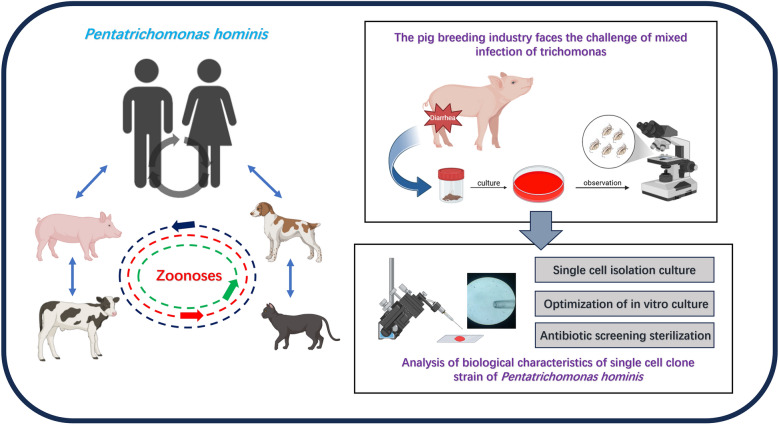

**Supplementary Information:**

The online version contains supplementary material available at 10.1186/s13071-025-06752-9.

## Background

Trichomonads are extracellular protozoan parasites characterized by the presence of multiple flagella, typically with four anterior flagella (AF) and one recurrent flagellum (RF) associated with an undulating membrane (UM). They possess hydrogenosomes (H) instead of mitochondria and lack cysts. They pose a significant health risk to both humans and mammals. A species of particular concern among trichomonads is *Pentatrichomonas hominis*, which primarily parasitizes the intestinal tract and is transmitted via the fecal-oral route [1]. While initially considered a commensal organism [2], recent studies suggest that it may be a causative agent of gastrointestinal and pulmonary diseases in both children and the elderly [3, 4]. A survey of Chinese gastrointestinal cancer patients revealed a high prevalence of *P. hominis* infection (41.5%) compared to that in the general population (9.1%), indicating a 6.75-fold increased risk of gastrointestinal cancer in infected individuals [5]. Additionally, this parasite is believed to be associated with systemic lupus erythematosus, irritable bowel syndrome, and rheumatoid arthritis in humans [6, 7]. In recent years, the epidemiology of *P. hominis* in various vertebrates has garnered considerable attention. For instance, this parasite has been detected in the feces of dogs afflicted with diarrhea in China and is now considered a potential cause of diarrhea in cats [8, 9]. The presence of *P. hominis* in common household pets, such as dogs and cats, also highlights the risk of zoonotic transmission. Studies on other mammals, including cattle, sheep, pigs, marmosets, sika deer, Rex rabbits, blue foxes, silver foxes, raccoon dogs, minks, and northeastern tigers, further demonstrate the high prevalence and diverse host range of this parasite [10–17].

In China, where pork is a staple of the diet, with over 70% of the country’s meat consumption being pork, it is crucial to understand the potential threats parasites pose to maintaining healthy pig breeding. The infected pigs showed diarrhea, emaciation and other symptoms, resulting in a decrease in daily weight gain and feed conversion rate. Severe infection can reduce the growth rate by 10%–30%, prolong the slaughter time, and increase the feed cost. Epidemiological surveys indicate that *P. hominis* infection rates in pigs are alarmingly high, reaching up to 24.1%, surpassing other trichomonad species such as *Tritrichomonas suis* and *Tetratrichomonas buttreyi* [12]. In 2014, Li et al. [18] identified a strain of *P. hominis* in a pig afflicted with diarrhea, and subsequent animal studies demonstrated this parasite’s ability to parasitize the cecum region of pigs and potentially act as a conditional pathogen. Therefore, *P. hominis* infection presents a significant risk to pig breeding and the pork industry in China.

Single-cell cloning is a widely employed technique in parasitology aimed at isolating parasite strains and ensuring their purity [19]. Previous studies have successfully isolated single-cell clones of *Tetratrichomonas gallinarum* and *Trichomonas gallinae* and optimized them for in vitro culture conditions using techniques such as micromanipulation [20–22]. These techniques have facilitated the study of parasite pathogenicity, epidemiology, and evolution. While it is effective in separating individual parasite cells, it may not directly address bacterial contamination issues. To mitigate bacterial contamination, additional measures such as the use of antibiotics may be necessary. However, to the best of our knowledge, there have been no reported efforts to establish single-cell clones of *P. hominis*.

The objective of this study was to isolate trichomonads from diarrheic piglet fecal specimens, purify them through *P. hominis* isolation and antibiotic selection to obtain a pure and sterile cell culture, and subsequently establish a stable in vitro culture system. Molecular biology, phylogenetic analysis, and morphological identification were employed to determine the genetic and cellular characteristics, thereby providing practical guidance and theoretical references for similar studies.

## Methods

### Isolation and cultivation of porcine trichomonads

Fecal samples were collected from a piglet afflicted with diarrhea in Xinxing, Guangdong. It is imperative to store fecal samples at room temperature for < 24 h from the time of excretion to the time of separation. Initially, 100 mg of feces was diluted in 10 ml PBS, and the resulting mixture was added to a modified Diamond medium containing trypticase (BBL), yeast extract, maltose, l-cysteine hydrochloride, and l-ascorbic acid, adjusted to a pH of 7.2, in a 1:9 ratio [23]. Additionally, 10% fetal bovine serum (FBS, Procell), penicillin (100 IU/ml), and streptomycin (100 μg/ml) were supplemented to the medium. The cultures were incubated under anaerobic conditions at 37 °C for 48 h. To maintain anaerobic conditions during incubation, we utilized a sealed C-31 culture bag (Mitsubishi Gas Chemical Co., Inc., Japan) along with AnaeroPack C-01 (Mitsubishi Gas Chemical Co., Inc, Japan). Following incubation, a portion of the culture suspension was examined under a light microscope using a 400 × field of view.

### Establishment of single-cell clonal cultures

*Method 1* To obtain a pure strain of porcine trichomonads, a mouth-controlled capillary single-cell isolation device was employed. This device consisted of a borosilicate glass capillary pipette featuring a slender filamentous tip, with a sterile plastic tube attached at the posterior end. The pipette was equipped with 100-μl and 1000-μl tips at each end and a 100-μl tip attached to the mouth pipette. After the trichomonad isolates had been mixed and cultured for 24 h to reach the logarithmic growth phase, limited dilution was performed until only one trichomonad could be seen in the field of view. Using the mouth-controlled capillary single-cell separation device, individual parasites were aspirated into a 96-well cell culture plate containing a complete culture medium; these steps were performed under observation using a LEICA DMi8 inverted microscope. The wells were then examined again to confirm the presence of a single parasite within each well. The culture system was incubated anaerobically at 37 °C for 48 h, and the growth of the parasites was monitored.

*Method 2* In this method, a sample from the mixed culture of parasitic isolates was taken for a hemocytometer plate count, and the mixed culture was diluted in a 1:2 dilution ratio. This dilution process continued until the number of protozoa reached approximately one parasite per 2 μl of culture. Specifically, 2 μl of the protozoa culture was transferred into a 96-well cell culture plate containing a complete culture solution. The plate was then observed under an inverted microscope to ensure that each well contained a single protozoan. Wells were carefully examined, and those containing no protozoa or more than one individual were excluded from further analysis to ensure true single-cell isolation. The culture system was incubated anaerobically at 37 °C for 48 h.

The viability of the protozoa was assessed by employing a modified subculture technique that involved alternating between full and half medium volume changes. The initial subculture was performed by replacing the entire volume of the old medium with an equal volume of fresh medium. In the subsequent subculture, only half of the old medium was replaced with a 1:1 mixture of fresh medium and the filtrate of the original trichomonad culture passed through a 0.22-μm filter. This filtrate contained secreted metabolites and pheromones from the original culture while removing the trichomonads themselves. The alternating pattern of full medium change and half medium change with the addition of the filtered original culture was maintained throughout the study to evaluate its impact on the survival and growth of the protozoa cultures.

### Culture system de-bacterization process

To obtain a pure protozoa culture and identify any contaminating or symbiotic bacteria, a drug-sensitive paper screening test was conducted using 16 types of susceptibility test discs purchased from Hangzhou Microbial Reagent Co., Ltd. The susceptibility test discs included penicillin, chloramphenicol, erythromycin, metronidazole, amoxicillin, ampicillin, ciprofloxacin, enrofloxacin, lincomycin, amphotericin B, florfenicol, cefoxitin, ceftriaxone, cefmetazole, cephalexin, and cefamandole. A 100-μl sample of the protozoa culture at a concentration of 1 × 10^4^ cells/ml was coated with thioglycolate fluid medium (FT) and placed on the susceptibility test discs with three replicates of each tablet used. Following a 24-h incubation period at 37 °C, the diameter of the inhibition circle was measured to identify antibiotics with a robust inhibitory effect. These antibiotics were then used in a drug pressure screening to establish a sterile culture system.

### Optimization of in vitro culture conditions for single-cell clonal isolates

To determine the most suitable culture medium for the growth of trichomonads, the following experiments were conducted. Four different media were selected: TYM medium, DMEM-F12, RPMI 1640, and Diamond medium. FBS, penicillin, and streptomycin were supplemented to the medium to provide essential nutrients and prevent bacterial contamination. Trichomonad cultures were cultured in vitro to the logarithmic growth stage and centrifuged at 3000 × *g* for 5 min, and the precipitates were collected. The precipitates were resuspended in 1 ml different media. After all counts had been obtained using a hemocytometer plate, the cells were inoculated at a density of 1 × 10^4^ cells/ml in a 24-well cell culture plate with pure medium, devoid of serum. Time gradient sample wells were set up with three replicates for each time point, and the culture system was incubated anaerobically at 37 °C using a sealed culture bag C-31 (Mitsubishi Gas Chemical Co., Inc, Japan) with AnaeroPack C-01 (Mitsubishi Gas Chemical Co., Inc, Japan). Hemocytometer plate counts were performed every 12 h, commencing from the time of inoculation, and the growth curve of porcine trichomonads with various culture media was plotted based on the count results.

To determine the most suitable serum types for the growth of porcine trichomonads, the following experiments were conducted. Six different types of serum (Gibco, BI, Cell-box, Fuheng, Procell, and Royacel) were used. These were divided into two groups, inactivated and non-inactivated, and added to the Diamond medium at a 10% supplementation rate; trichomonads were inoculated at a concentration of 1 × 10^4^ cells/ml. The culture system was incubated anaerobically at 37 °C for 72 h. Hemocytometer plate counts were performed after the incubation period, and three replicates of each type of serum were employed.

To compare the effects of different inoculum amounts on the in vitro culture characteristics of porcine trichomonads, the following experimental procedures were conducted. Trichomonads were cultured until they reached the logarithmic growth phase. The precipitate was collected by centrifugation at 3000 × *g* for 5 min, and the precipitate was then resuspended in 1 ml Diamond medium. After cell counts had been obtained using a blood cell counting plate, the cells were inoculated in 24-well cell culture plates at three different inoculum levels: 1 × 10^6^ cells/ml, 1 × 10^5^ cells/ml, and 1 × 10^4^ cells/ml. Time gradient sample wells were set up with three replicates for each point. The culture system was placed under anaerobic conditions at 37 °C. Hemocytometer plate counts were performed every 24 h, commencing from the time of inoculation, and the in vitro culture growth curve of porcine trichomonads was plotted based on the count results.

### DNA extraction, PCR, and sequence analysis for single-cell clonal isolates

Trichomonads were cultured until they reached the logarithmic growth phase. The cells were subsequently collected by centrifugation at 3000 × *g* for 5 min and washed twice with phosphate buffer solution (PBS). Genomic DNA was extracted from the cells using the Trelief^™^ Animal Genomic DNA Extraction Kit (Tsingke Biotechnology Co., Ltd) following the manufacturer’s instructions. The extracted DNA was used as a template for PCR amplification of the trichomonads’ 18S rRNA gene [24], ITS1-5.8S rRNA-ITS2 gene [25], and EF-α gene [26], using the primers listed in Table 1. The PCR reaction conditions were set as previously reported [27]. The resultant PCR products, including the 18S rRNA gene/339 bp, ITS1-5.8S rRNA-ITS2 gene/364 bp, and EF-1α gene/782 bp, were purified from agarose gels using Hipure Gel Pure DNA Mini Kit (Magen Biotechnology, China). These purified DNA fragments were then ligated into the pMD18-T Simple Vector (TaKaRa, Japan), transformed into DH-5α cells (Tsingke Biotechnology Co., Ltd.), and subsequently sequenced by Guangzhou IGE Biotechnology Company.

**Table 1:** Primer sequence for identification of *Pentatrichomonas hominis*

Gene	Primers	Sequences (5′–3′)	Reference
18S rRNA	Th3	TGTAAACGATGCCGACAGAG	[8]
	Th5	CAACACTGAAGCCAATGCGAGC	
ITS1-5.8S rRNA-ITS2	NC5	GTAGGTGAACCTGCGGAAGGATCATT	
	NC2	TTAGTTTCTTTTCCTCCGCT	
EF-1α	ef-F	GACTTCATCAAGAACATGATCAC	
	ef-R	GCGATGTGAGCTGTGTGGC	

The nucleotide sequences obtained were aligned with their respective reference sequences using ClustalX 1.81 software. The aligned sequences were then imported into MEGA X software to assess the best-fit model and parameters based on Akaike. The sequences were subjected to phylogenetic analysis using the Neighbor-Joining algorithm with 1000 bootstrap replications and the best-fit model. The purpose of this analysis was to evaluate the relationship between the trichomonads isolated in this study and those described in previous studies.

### Microscopic observation of single-cell clonal isolates

#### Light microscopy

After being cultured in vitro until reaching the logarithmic growth phase, porcine trichomonads were centrifuged at 3000 × *g* for 5 min and subsequently resuspended in 1 ml Diamond medium for sedimentation. The resulting suspension was uniformly applied to slides and fixed by air drying. Parasite staining was performed using modified Giemsa stain (Beyotime Biotechnology) [28] and Diff-Quik stain (SenBeiJia Biological Technology Co., Ltd.) [22] in accordance with the manufacturer’s instructions. All smears were then examined under a microscope and documented through photography.

#### Scanning electron microscopy (SEM)

Trichomonad samples were cultured in vitro up to the logarithmic growth phase and then subjected to centrifugation at 3000 × *g* for 5 min. The cellular precipitates were collected and washed twice with PBS. The samples were then pre-fixed using 2.5% glutaraldehyde, followed by removing excess fixative by washing with PBS. Next, the samples were post-fixed using 1% OsO_4_ for 3 h and subjected to gradient dehydration and critical point drying using varying alcohol concentrations. Finally, the dried samples were mounted on a sample stage, examined, and documented via photography using a Hitachi S-3400N-II scanning electron microscope (Hitachi, Japan).

#### Transmission electron microscopy (TEM)

Trichomonad cultures were expanded and prepared for transmission electron microscopy (TEM) analysis. The samples were fixed, dehydrated following the methodology delineated in the SEM section, and subsequently infiltrated with ethylene oxide and Spurr resin. The treated samples were then embedded in fresh epoxy resin and polymerized in an oven. Finally, ultra-thin sections of the cured resin blocks were obtained and mounted onto copper grids, and the specimens were observed and photographed using a Hitachi HT7700 transmission electron microscope (Hitachi, Japan).

#### Statistical analysis

In this study, the data were initially organized using Microsoft Excel and subsequently plotted and subjected to statistical analysis using GraphPad Prism8 software, employing ANOVA for multiple comparison variance analysis. Significance levels are indicated using asterisks, with * indicating *p* < 0.05, *** indicating *p* < 0.0005, and **** indicating *p* < 0.0001.

## Results

### Isolation and in vitro culture of porcine Trichomonads from fecal samples

**Fig. 1:**
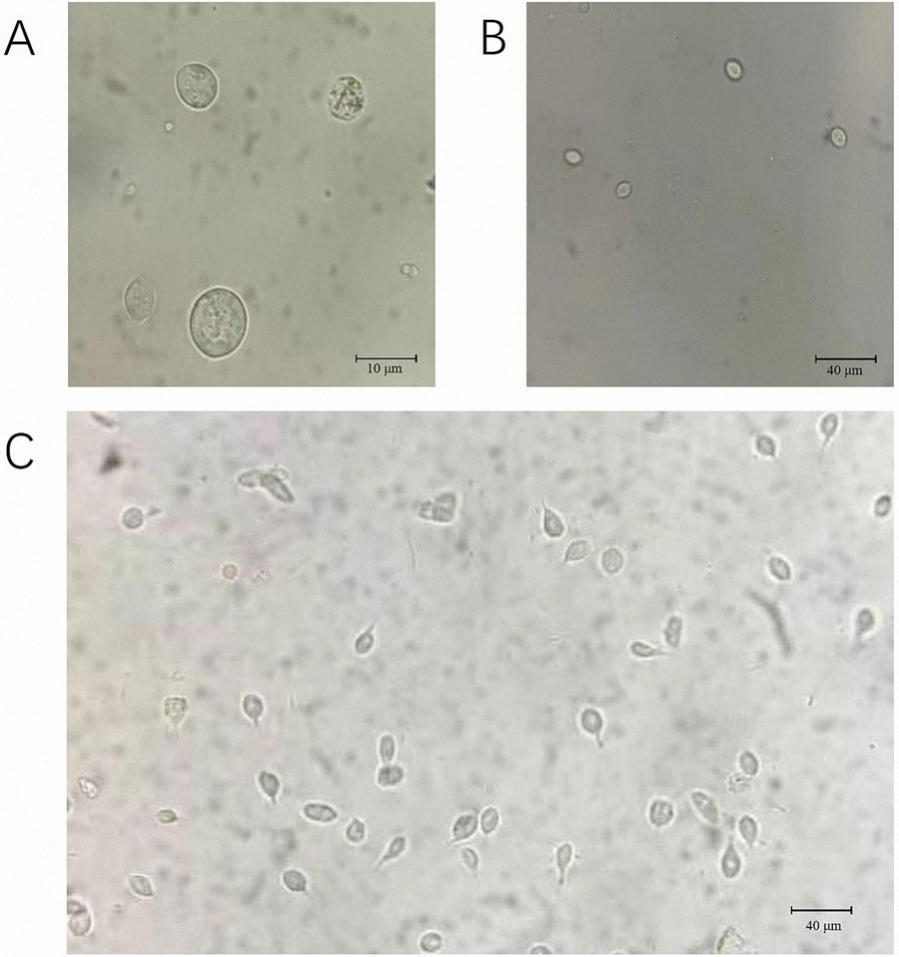
Trichomonad isolates in direct microscopy: **A** 1000 ×, **B** 400 ×, **C** 400 ×

After isolating cultures from fecal samples and incubating them anaerobically at 37 °C for 48 h, numerous fast-moving, pear-shaped protozoa were observed under the microscope. These motile protozoa exhibited morphological characteristics and motility consistent with those previously described for trichomonads. These organisms thrived under anaerobic conditions at 37 °C and were subcultured every 2–3 days (Fig. 1).

### Optimization of porcine Trichomonad single-cell clonal isolates for enhanced proliferation and survival rates

**Fig. 2:**
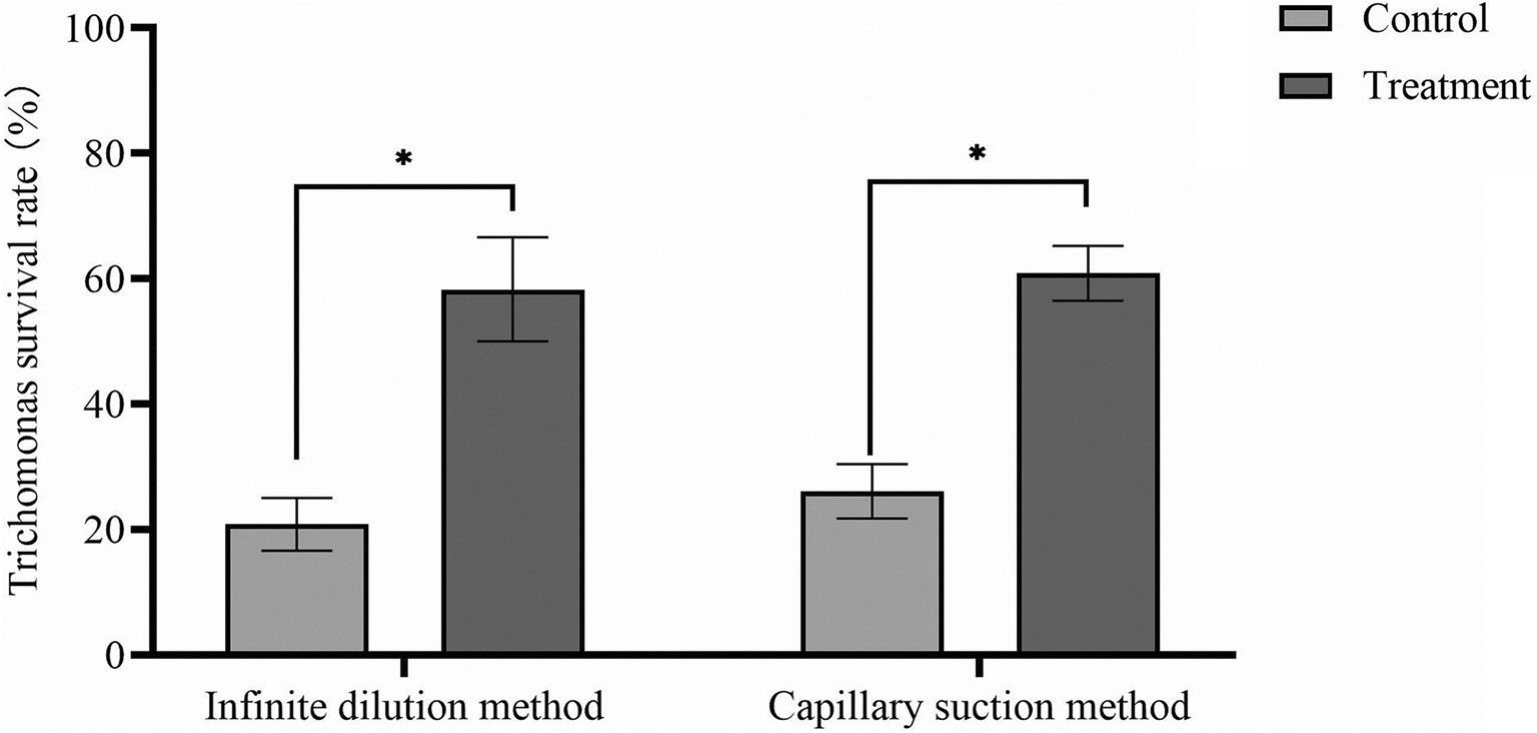
Comparison of the survival rate of porcine trichomonads using different isolation and culture methods. The methods used in this study include the infinite dilution method and capillary suction method. Control represented complete medium change culture, and treatment represented half medium change culture (compared with control, treatment showed a significant difference, two-way ANOVA, *P* < 0.05)

Employing different conditions for porcine trichomonad isolation and culture, the samples were incubated in vitro for 48 h. The isolated trichomonads exhibited characteristic morphological features as previously described [18, 44]: pear-shaped or ovoid cells measuring 7–10 μm in length, with active motility driven by five AFs and one RF. The UM was clearly visible along the cell body, and the organisms displayed rapid, jerking movements typical of trichomonads. Under the optical microscope, 12 of the 54 wells containing one protozoan obtained by the infinite dilution method survived after in vitro culture, with a positive rate of 22.2%. In contrast, the capillary pipette method yielded 23 positive wells out of 54 single trichomonad isolates, indicating a 42.6% positive rate (Table 2). However, when the late stage of protozoa proliferation was reached after 48 h in vitro culture, some strains showed undergrowth or delayed proliferation in both modes of transmission by completely changing the medium. To tackle this challenge, a modification was introduced in the form of a half-quantity liquid exchange culture approach. Here, the medium was divided into two halves: one containing the filtrate of the original trichomonad culture passed through a 0.22-μm filter, which retained the metabolites or pheromones, and the other comprising fresh medium during each passage. Notably, this adjustment aimed to provide a conducive environment for the survival and growth of porcine trichomonad cultures. Subsequent analysis revealed that this half-quantity liquid exchange culture method significantly enhanced the survival rate of porcine trichomonad cultures (Fig. 2).

**Table 2:** Isolation results of single-cell cloning porcine trichomonads

Method	Positivity	Negative	Positive rate	Total
Infinite dilution method	12	42	22.2%	54
Capillary suction method	23	31	42.6%

### Bacterial elimination in porcine Trichomonad single-cell clonal isolates

**Fig. 3:**
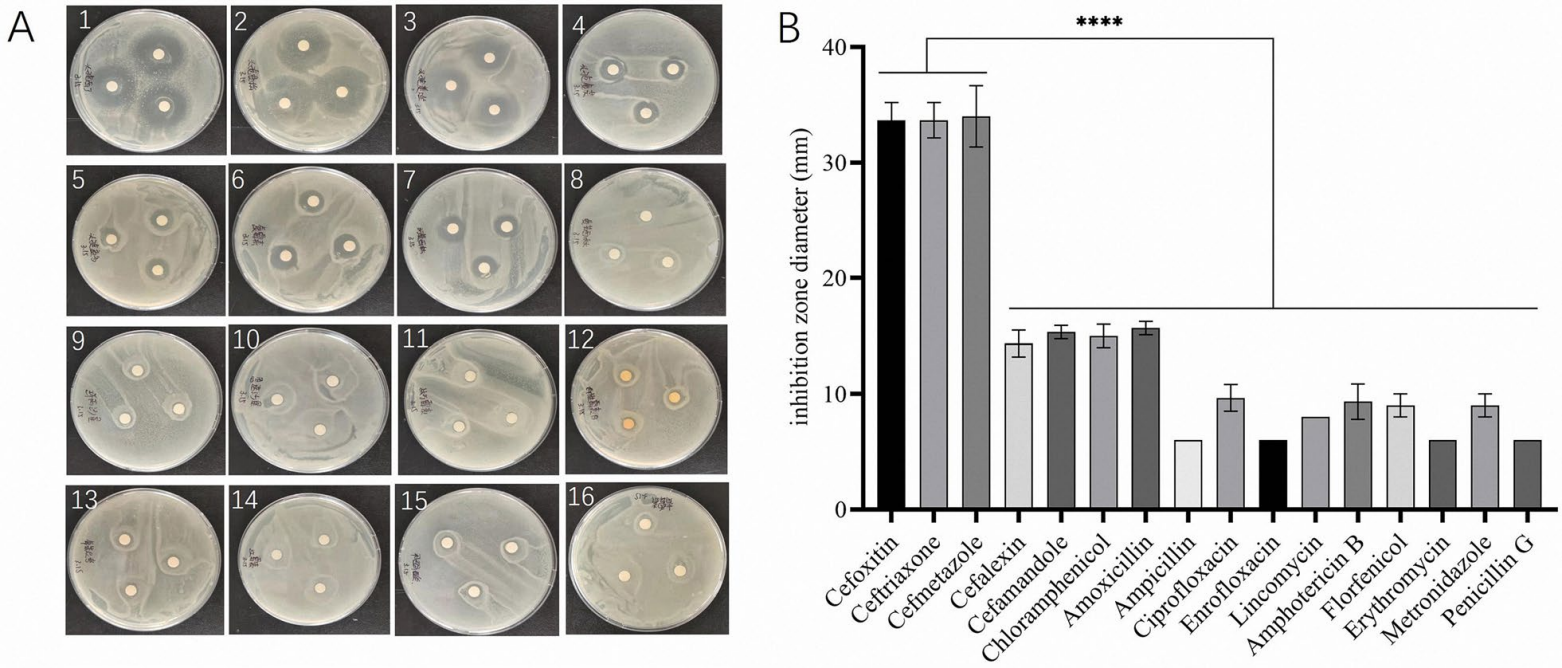
Screening results of drug sensitivity using the drug-sensitive slips method. The antibacterial-circle experiment involved 16 types of drugs (**A**), including cefoxitin (A1), ceftriaxone (A2), cefmetazole (A3), cefalexin (A4), cefamandole (A5), chloramphenicol (A6), amoxicillin (A7), ampicillin (A8), ciprofloxacin (A9), enrofloxacin (A10), lincomycin (A11), amphotericin b (A12), florfenicol (A13), erythromycin (A14), metronidazole (A15), and penicillin G (A16). The diameter of each drug’s inhibition zone in the inhibition zone test was statistically analyzed (**B**). The error bars represent the standard deviation of three biological replicates for each condition. Cefoxitin, ceftriaxone, and cefmetazole showed significant antibacterial activity against other antibiotics (one-way ANOVA, *P* < 0.0001 among all groups)

Bacteriological analysis of porcine trichomonad cultures revealed the presence of a sepsis-associated pathogen originating from the intestinal microbiota. Sensitivity testing using antibiotic-impregnated discs revealed that cefoxitin, ceftriaxone, and cefmetazole had the highest inhibition zones, measuring 33.7 mm, 33.7 mm, and 34 mm in diameter, respectively. Remarkably, all cephalosporins exhibited greater inhibition compared to other antibiotic classes (Fig. 3). Based on these findings, cefmetazole, which exhibited the most potent bacterial inhibition effect, was selected for further investigation in this study. Drug pressure screening confirmed that cefmetazole was effective in eradicating the commensal bacteria at a concentration of 100 μg/ml while preserving the growth of porcine trichomonads.

### Optimization of in vitro culture conditions for porcine Trichomonad single-cell clonal isolates

**Fig. 4:**
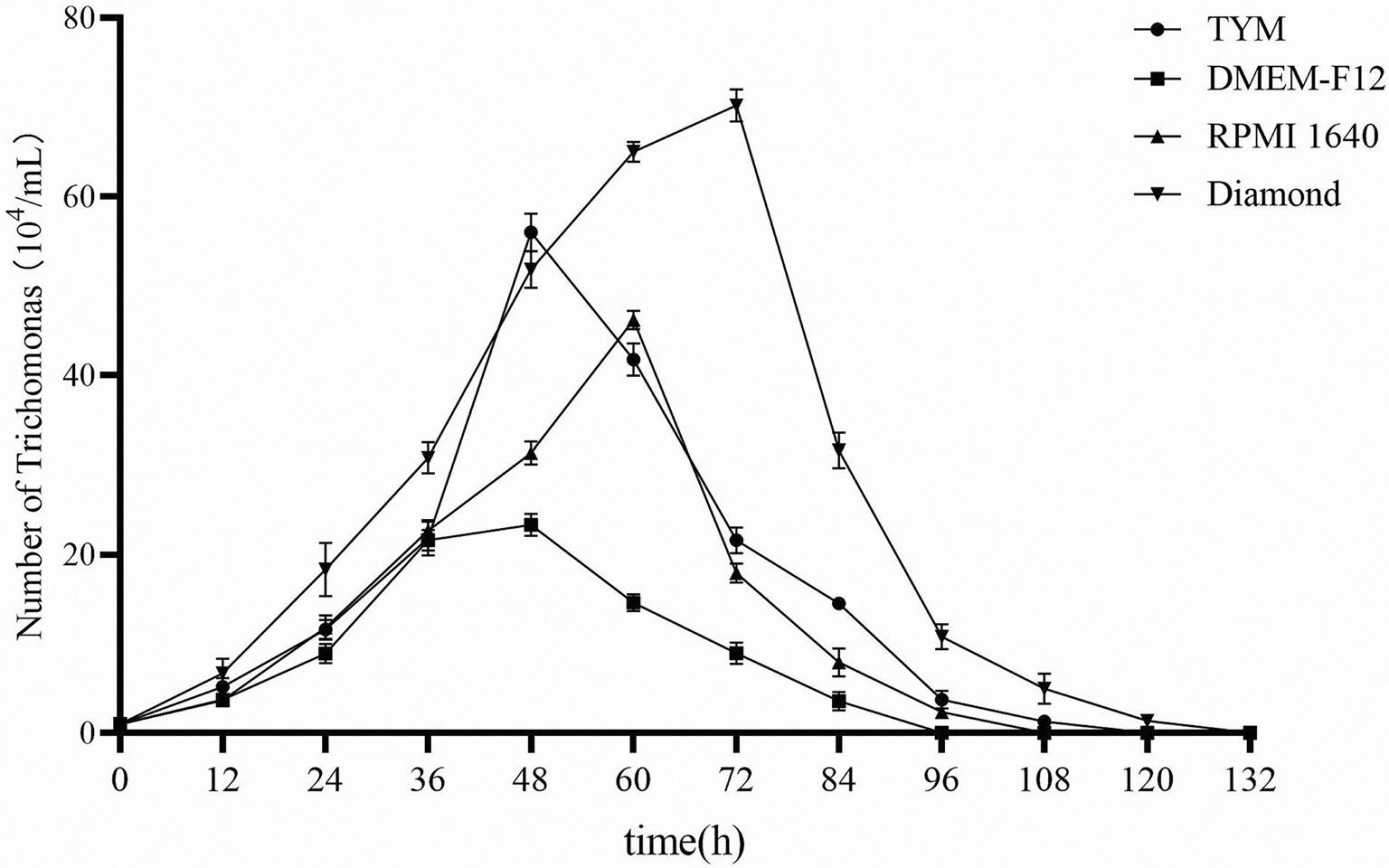
Growth curve of porcine trichomonads isolated in different culture media. The media used in this experiment included TYM medium, DMEM-F12, RPMI 1640, and Diamond medium

Our explorations of in vitro expansion culture conditions for porcine trichomonads revealed that the Diamond medium was the most effective for initial inoculum densities of 1 × 10^4^ cells/ml. Within the initial 72 h, the density of porcine trichomonads exhibited substantial growth, reaching a maximum concentration of 7.02 × 10^5^ cells/ml. In comparison, those cultured using the RPMI 1640 medium reached a maximum density of 4.62 × 10^5^ cells/ml at 60 h; those cultured using the TYM medium and DMEM-F12 medium attained their highest densities at 48 h, with 5.60 × 10^5^ cells/ml and 2.33 × 10^5^ cells/ml, respectively (Fig. 4).

**Fig. 5:**
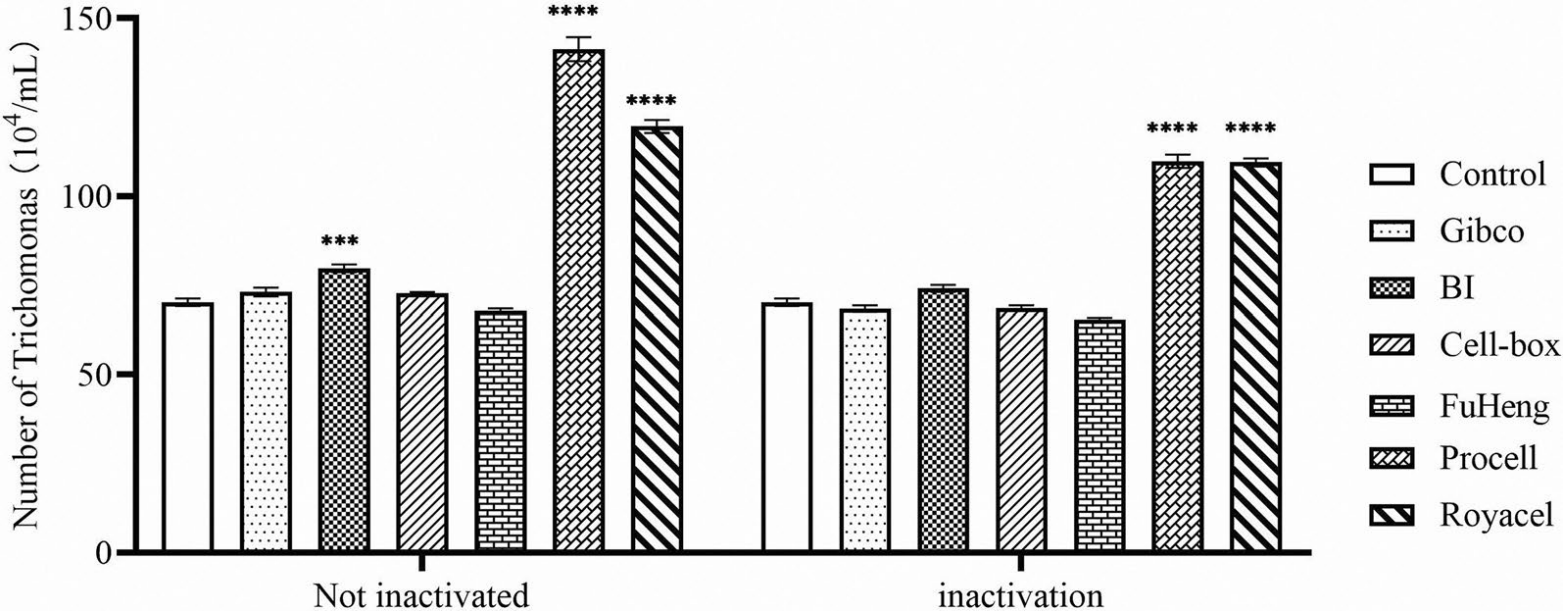
Effect of different serum brands on the sterile culture of porcine trichomonads. The serums used in this study included Gibco, BI, Cell-box, Fuheng, Procell, and Royacel (two-way ANOVA, *significant difference compared with the blank control group, ***P < 0.001; ****P < 0.0001)

The influence of different brands of serum on porcine trichomonad density after 72 h of incubation was investigated, both with and without inactivation treatment. Our findings revealed the groups introduced to Procell and Royacel serum exhibited significantly elevated porcine trichomonad density compared to the group without serum supplementation. Furthermore, when inactivated BI serum was introduced, the density of porcine trichomonads at 72 h was significantly higher than in the non-serum group. The addition of inactivated serum of other brands exhibited a slight inhibitory effect on porcine trichomonad growth compared with normal serum, but this difference was not statistically significant (Fig. 5).

**Fig. 6:**
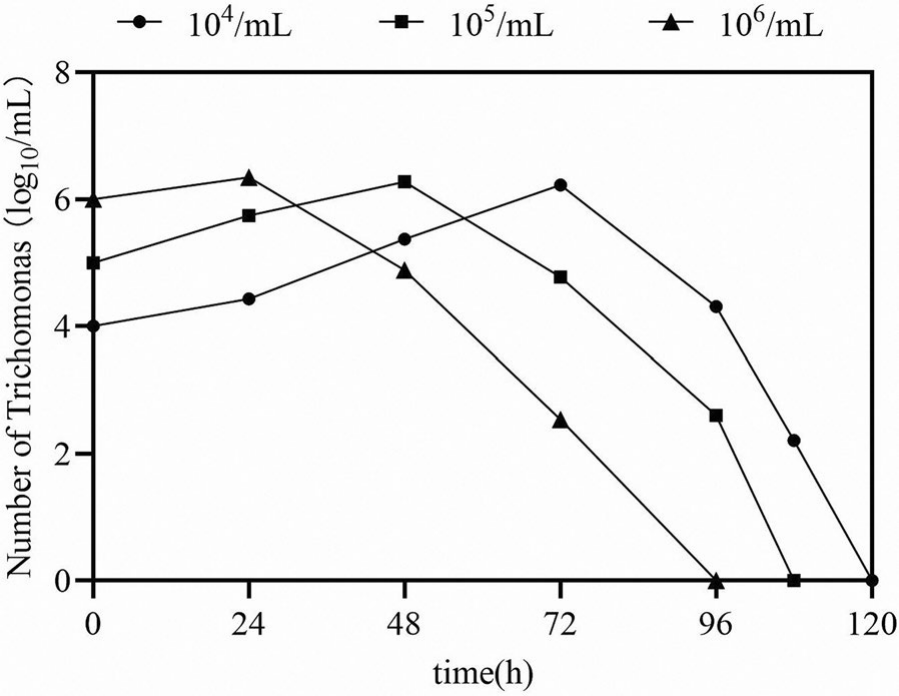
Growth curve of porcine trichomonads at different inoculation cell densities

The growth curves of porcine trichomonads at different inoculum densities showed that at an initial inoculum density of 1 × 10^6^ cells/ml, the highest density of porcine trichomonads was approximately 2.21 × 10^6^ cells/ml at the 24-h mark. For an initial inoculum density of 1 × 10^5^ cells/ml, the highest density of porcine trichomonads was approximately 1.88 × 10^6^ cells/ml, observed at the 48-h mark. Moreover, with an initial inoculum density of 1 × 10^4^ cells/ml, the highest density of porcine trichomonads, approximately 1.68 × 10^6^ cells/ml, was recorded at 72 h. After the highest density of porcine trichomonads had been attained, the population experienced a rapid decline across all inocula (Fig. 6).

### Sequence cloning and phylogenetic analysis of porcine Trichomonad single-cell clonal isolates

**Fig. 7:**
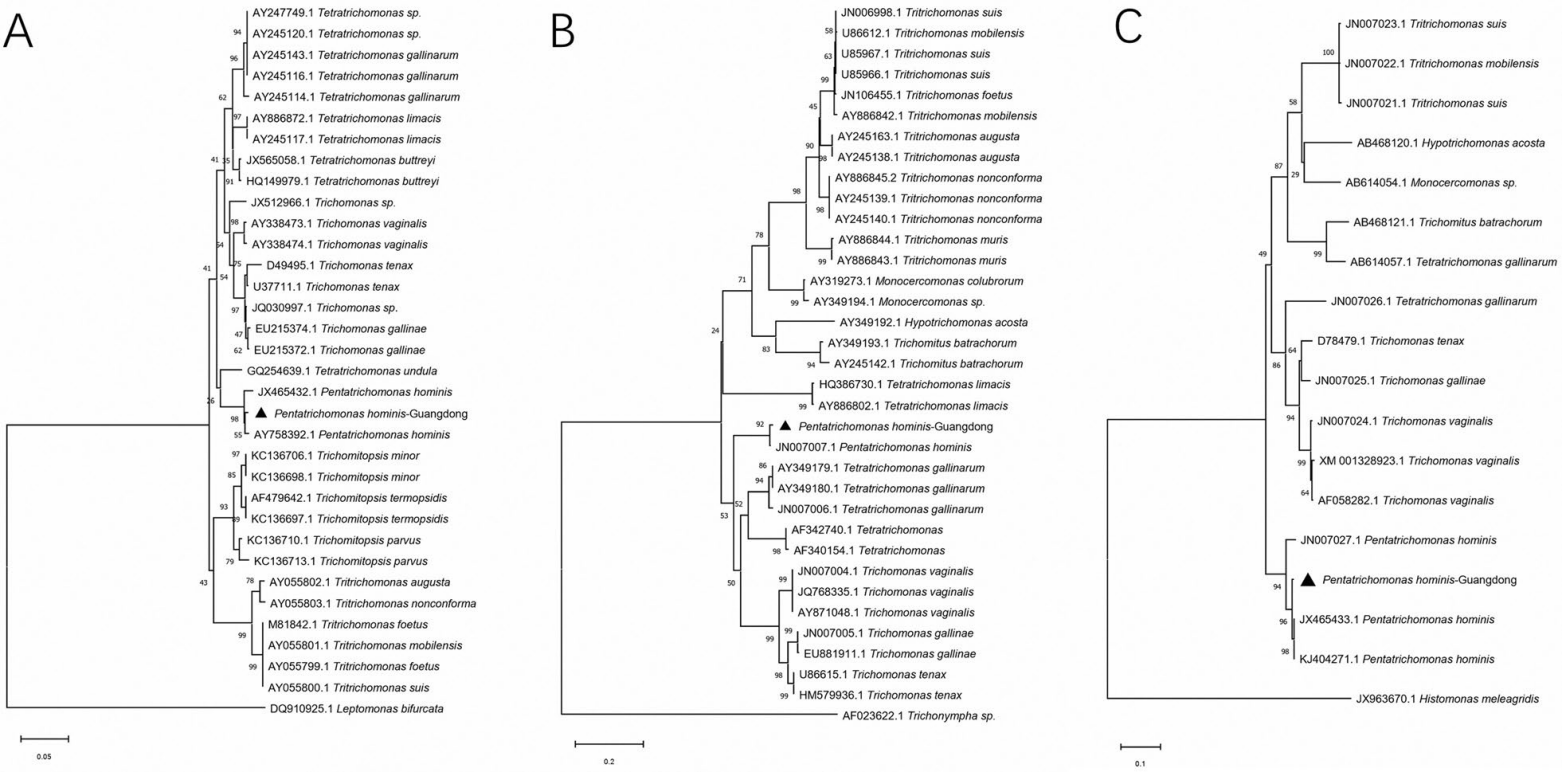
Phylogenetic tree of porcine trichomonads compared with other trichomonads. Three genes were used to construct the phylogenetic tree: the 18S rRNA gene (**A**), ITS1-5.8S rRNA-ITS2 gene (**B**), and EF-1α gene (**C**). Bayesian posterior probabilities > 0.7 or bootstrap percentages > 50% from 1000 replicates are shown. The outgroup used for rooting trees included *Leptomonas bifurcata* (accession no. DQ910925.1), *Trichonympha* sp. (accession no. AF023622.1), and *Histomonas meleagridis* (accession no. JX963670.1)

After PCR amplification and sequencing, we successfully cloned the 339-bp 18S rRNA gene, the 364-bp ITS1-5.8S rRNA-ITS2 gene, and the 782-bp EF-α gene from the pure trichomonad isolate. Subsequent sequence alignment analysis revealed that the 18S rRNA gene of the isolate shared a remarkable 99.4% sequence identity with the reference *P. hominis* isolate (GenBank accession no. AY758392), with merely two base mismatches. The ITS1-5.8S rRNA-ITS2 gene sequence of the isolate was completely identical to the reference *P. hominis* isolate (GenBank accession no. KJ404270). However, the EF-α gene of the isolate exhibited 26 single-nucleotide polymorphisms (SNPs) compared to the reference *P. hominis* isolate (GenBank accession no. JN007027), resulting in a sequence identity of 96.7%. Phylogenetic analysis revealed that the isolate was closely related to the human pathogenic *P. hominis* (Fig. 7).

### Cell morphology and microstructural characteristics of porcine Trichomonad single-cell clonal isolates

**Fig. 8:**
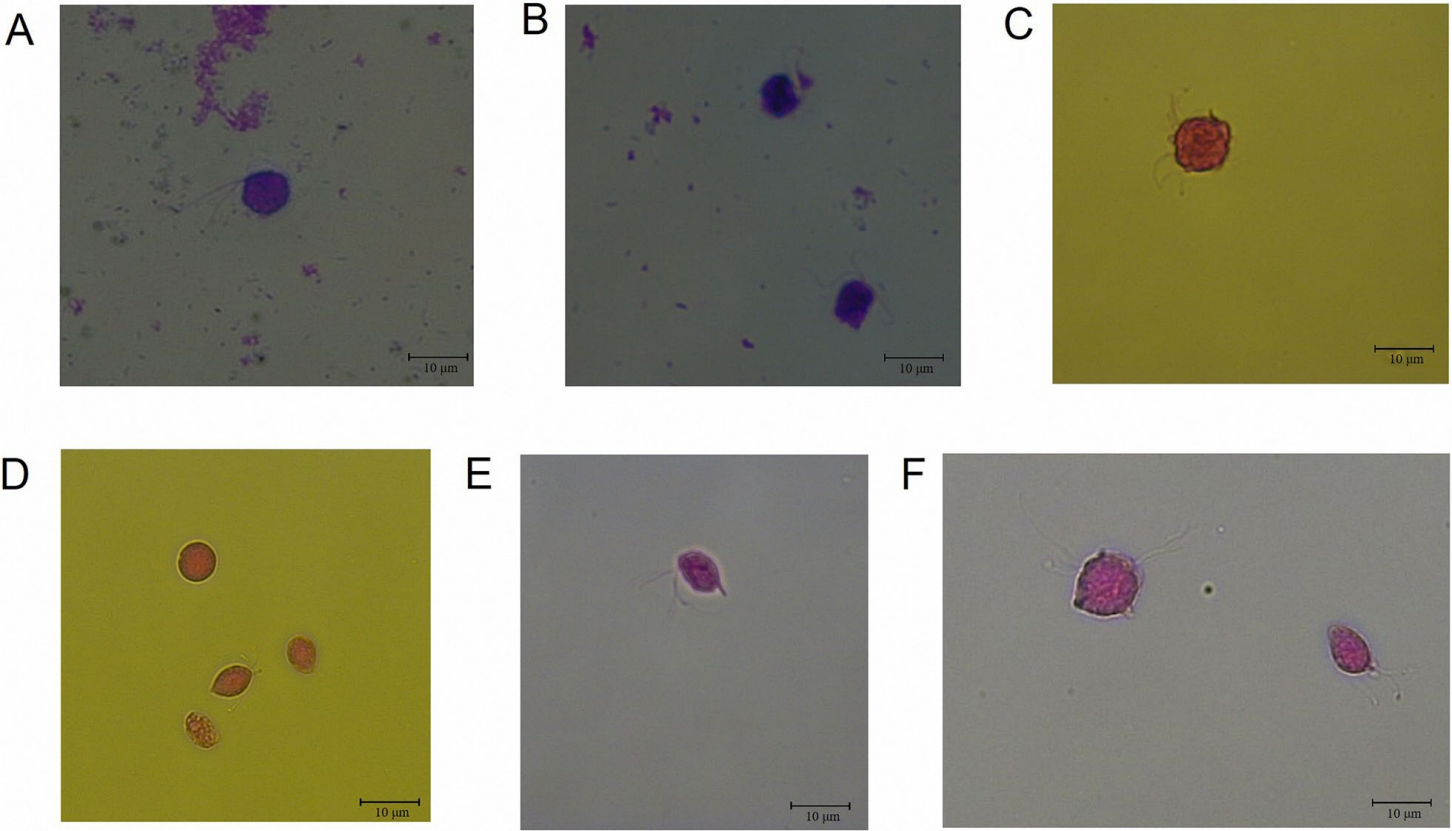
Morphological observation of porcine trichomonads using different staining methods in direct microscopy at 1000 × magnification. The methods employed were the modified Giemsa staining method (**A**–**B**) and Diff-Quik staining method (**C**–**F**)

The trichomonads in the trophozoite stage were observed using light microscopy following modified Giemsa staining (Fig. 8A–B) and Diff-Quik staining (Fig. 8C–F). The trichomonads exhibited a pear-shaped or ovoid morphology, with approximate dimensions ranging between 7 and 10 μm. Both staining techniques revealed the distinctive flagella features of the trichomonads without any significant disparities. The pseudocyst period of the trichomonads was also observed (Fig. 8D).

**Fig. 9:**
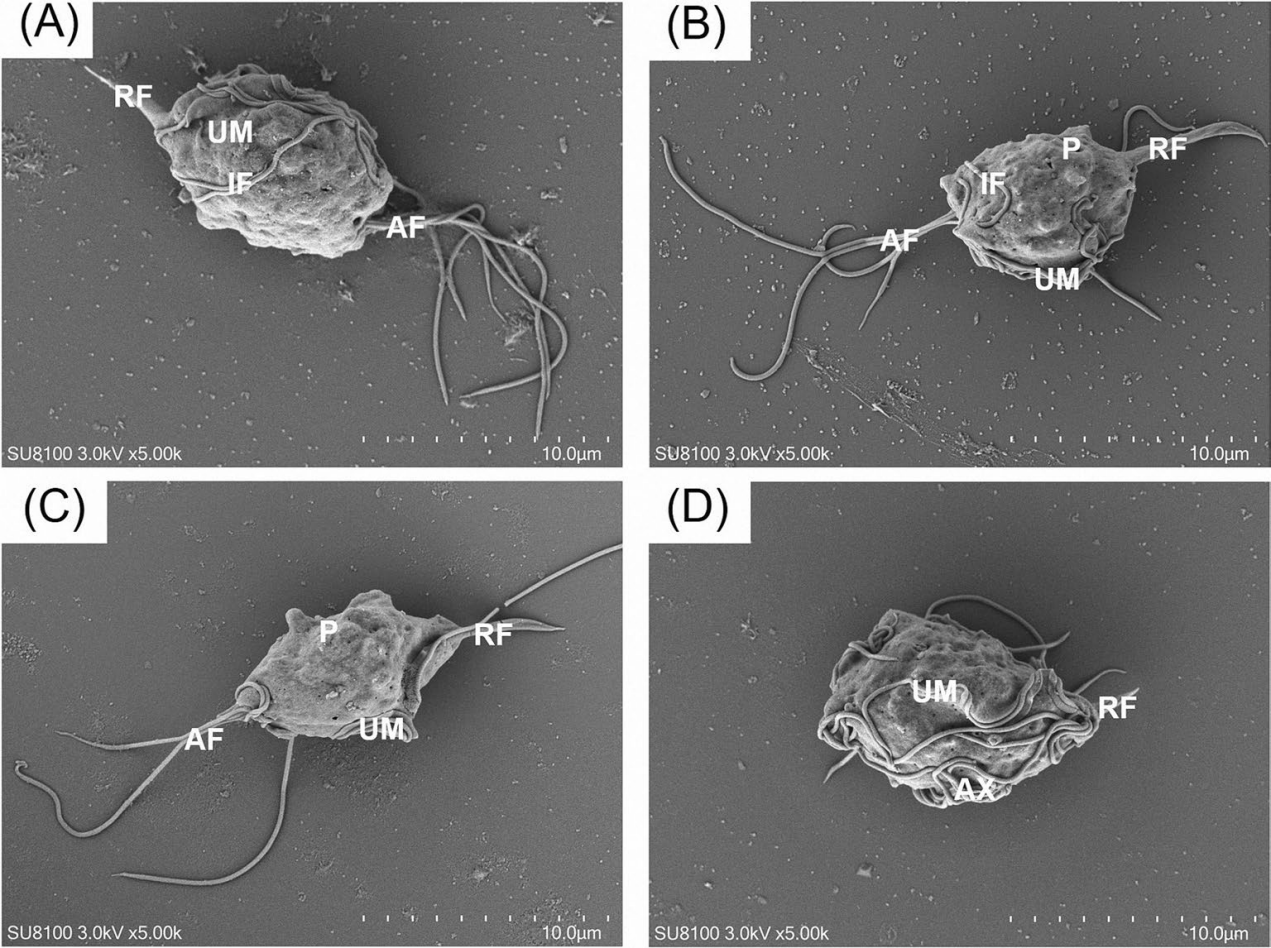
Observation of *Pentatrichomonas hominis* under scanning electron microscope. SEM micrographs of *P. hominis* (**A**–**D**) demonstrated *P. hominis* has *AF* anterior flagella, *UM* undulating membrane, *RF* recurrent flagellum, *P* pelta, and *AX* axostyle

The ultrastructure of the trichomonad culture was examined using scanning SEM and TEM. Our examinations confirmed that the culture showed characteristics similar to those of *P. hominis*, consistent with previous studies. Under SEM observation, the trophozoites of the trichomonads had dimensions of approximately 7 μm × 5 μm, featuring one RF and four AF (Fig. 9). The flagella were intertwined with each other. The UM and pelta (P) (the demarcation between the AF and RF) were visible along the cell extension; however, the cytosol was not smooth and had a wrinkled appearance. These structural features differed from those observed in *Trichomonas* spp. infections such as *T. suis* and *T. buttreyi*.

**Fig. 10:**
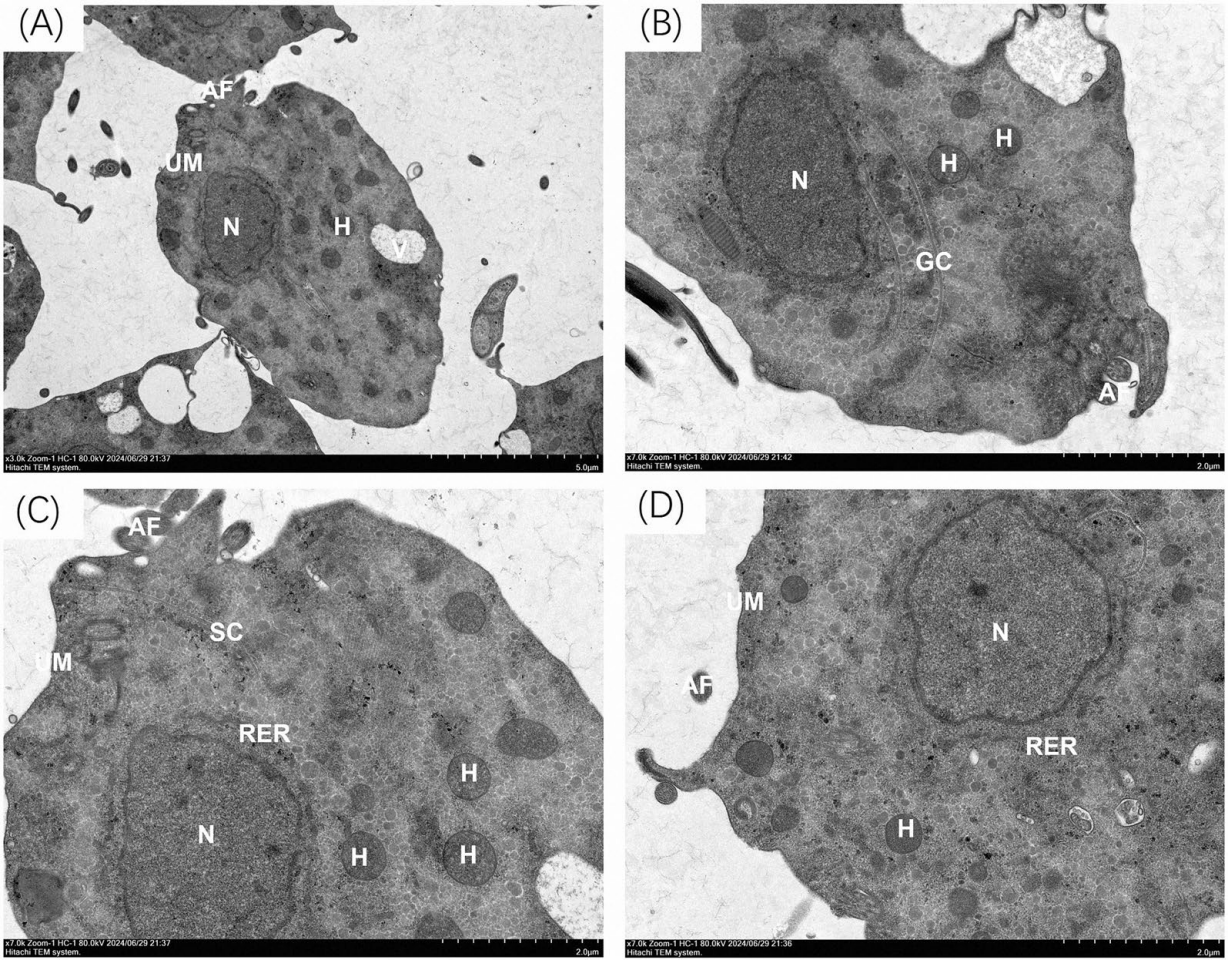
Observation of *Pentatrichomonas hominis* under a transmission electron microscope. TEM micrographs of *P. hominis* (**A**–**D**) demonstrated *P. hominis* has *SC* striated costa, *UM* undulating membrane, *N* nucleus, *H* hydrogenosomes, *V* vacuoles, *RER* rough endoplasmic reticulum, *GC* Golgi complex, *AF* anterior flagella

The internal structure of trichomonads in the trophozoite stage was observed using TEM (Fig. 10). A prominent nucleus (N) was located in the mid-anterior region within the cytosol, and varying numbers of hydrogenosomes were dispersed throughout the cell cultures. Moreover, food vacuoles (V) were located at the edge of the cytosol. Additionally, organelles such as the striated costa (SC), UM, rough endoplasmic reticulum (RER), and Golgi complex (GC) exhibited integrated regions with varying electron density. This ultrastructural configuration was consistent with the electron microscopic characteristics previously reported for *P. hominis* [18].

## Discussion

*Pentatrichomonas hominis* is a potential zoonotic pathogen known to cause gastrointestinal syndrome in humans [3]. It has also been detected in dogs, cats, and pigs afflicted with diarrhea, elevating the risk of zoonotic transmission [29, 30]. Moreover, recent studies indicate a high prevalence of *P. hominis* infection in patients with gastrointestinal cancers, suggesting its zoonotic pathogenicity [5]. Despite these findings, there is limited knowledge regarding its pathogenicity and infection mechanisms, especially in pigs, which constitute a significant food source in China.

One of the challenges in studying parasites such as *P. hominis* is the occurrence of mixed infections or co-infections with bacteria during the isolation and purification process [31]. In the present study, the fecal samples were identified by PCR before the isolation of the strains. The results showed that the samples contained *P. hominis*, *T. buttreyi*, and *T. suis* (data not shown). To gain a better understanding of the pathogenicity and genetic evolution of these parasites, it is crucial to establish pure and sterile cultures. Conventional methods for the separation and purification of parasites include micromanipulation and limited dilution techniques [32–34]. In this study, we developed a novel mouth-controlled technique for isolating monoclonal trichomonad cells, which offers a simpler and more cost-effective alternative to micromanipulation while ensuring higher precision than the limited dilution method. Additionally, we improved the survival rate of trichomonads in culture by implementing a half-volume medium exchange at a later stage, which may preserve specific metabolites or signaling molecules that facilitate information exchange among trichomonads [35]. This innovative approach enabled us to establish a stable and sterile culture of *P. hominis*, overcoming previous challenges encountered in culturing this parasite [36]. Our study marks the first report of a single-cell cloned and axenic culture of *P. hominis*, although there have been prior reports of single-cell cloning and purification of *T. gallinarum* and *T. gallinae* [20, 21].

The growth curve measurements of the in vitro culture align with the findings of previous studies [8]. In this study, the issue of bacterial contamination during *P. hominis* culture was successfully resolved through antibiotic selection and optimization of in vitro culture conditions. This resulted in the establishment of a stable and aseptic in vitro culture system. The establishment of a pure and sterile culture of *P. hominis* is crucial for understanding its pathogenicity, host range, and genetic evolution. This is especially important in light of its high prevalence in both human and animal populations, highlighting its potential as a zoonotic pathogen. Previous studies have shown that the growth of trichomonads is significantly influenced by media composition, particularly the supplementation of serum, which serves as a crucial nutrient source [37]. While the addition of 10% serum is widely accepted as optimal, there has been limited systematic investigation into the effects of different serum types (horse serum, fetal bovine serum, and sheep serum) on trichomonad growth [18, 23, 38]. Furthermore, the necessity of serum inactivation, traditionally performed to remove complement components, remains questionable for fetal bovine serum (FBS) because of its inherently incomplete complement system. In addition to media composition and serum considerations, inoculum density has been identified as a critical factor affecting trichomonad growth and proliferation rates. Our findings suggest that culture conditions should be optimized specifically for different trichomonad isolates. Through systematic optimization of culture media components, serum type selection, and inoculation parameters, we successfully established a stable in vitro culture system for porcine-derived *P. hominis*. This system utilizes a modified Diamond medium supplemented with 10% non-inactivated Procell FBS. The culture can be maintained without antibiotics or with standard antibiotic supplementation (e.g., penicillin-streptomycin) as per conventional practices.

Light microscopy serves as a supplementary technique for the detection of trichomonads [39]. Accurate identification of pathogenic species is crucial for the diagnosis, treatment, and detection of diseases. Microscopic examination is considered the most direct method for diagnosing parasitic pathogens. Traditionally, trichomonad infections have been diagnosed by the microscopic examination of fresh fecal samples or fecal cultures [40]. However, different trichomonad species share similar sizes, shapes, and motility, making it challenging to distinguish them under light microscopy [41]. Molecular biology methods, such as PCR, offer high sensitivity, specificity, and speed, enabling more accurate genetic typing by amplifying specific nucleotide sequences or genetic markers of trichomonads. This technology has also been established as an effective tool in veterinary parasitology for diagnosis, quantitative analysis, and standardization of gene expression [42]. Optimized PCR detection methods are more sensitive and specific compared to traditional optical microscopy and can be employed for trichomonad detection [43]. Molecular biology methods are better suited for the evolutionary genetic classification of trichomonad species [26]. In the present study, we confirmed the trichomonad isolates were *P. hominis* by amplifying the nucleotide sequences of three genes: the 18S rRNA gene, ITS1-5.8S rRNA-ITS2 gene, and EF-α gene, followed by phylogenetic analysis. These findings were further substantiated by morphological observations using both light microscopy and electron microscopy.

Using light microscopy, we observed numerous rapidly moving pear-shaped parasites, which exhibited a size and motility similar to trichomonads [44]. We also identified trichomonads in the pseudocyst stage, which displayed a morphology similar to that of *Tritrichomonas foetus* [45]. The pseudocyst stage represents a reversible self-protective mechanism triggered by unfavorable external stimuli, a process that has been well-documented in several other trichomonad species. For instance, time-lapse microscopy studies have directly captured the transformation from pseudocyst to trophozoite forms in *T. foetus* [46, 47] and *Trichomonas vaginalis* [48], demonstrating the dynamic and reversible nature of this process. Although we did not observe the pseudocyst-to-trophozoite transition for *P. hominis* in the current study, the close evolutionary relationship and shared morphological features among these trichomonads strongly suggest that *P. hominis* pseudocysts also revert to the trophozoite stage under favorable conditions. This reversible transformation is thought to be a conserved adaptation enhancing the transmission and survival of trichomonad parasites in changing environments [47]. Further studies using time-lapse microscopy will be valuable to directly confirm the pseudocyst-trophozoite transition in *P. hominis*.

Otherwise, SEM allowed us to examine the detailed morphology of the trichomonad isolate, including the complex arrangement of flagella at the posterior end of the trophozoite, AF, and a distinctive RF located at the front end of the parasite, which is a characteristic feature of *P. hominis*. The number of flagella observed under SEM differed from those reported for *T. foetus* [49]. We also examined the UM, costa, and axostyle of the trichomonads. The UM of this isolate was notably longer than that of other trichomonads and exhibited a feature similar to *P. hominis* and *T. vaginalis* [50]. The internal ultrastructure of the isolated trichomonads was also investigated using TEM. The nucleus of the isolate was oval and located at the front of the insect. In addition to the RER and GC, food vacuoles and hydrogenosomes, which are common structures in other trichomonads, were detected [51].

This study provides valuable insights into the cultivation and characterization of *P. hominis*, contributing to the broader understanding of *Trichomonas* spp. Our findings align with previous studies on other trichomonads, such as *T. vaginalis* and *T. foetus*, in terms of growth patterns and morphological features [52, 53]. However, we observed unique characteristics in *P. hominis*, particularly in its flagellar arrangement and UM structure, which distinguish it from other species. These differences underscore the importance of species-specific research within the family Trichomonadidae. Despite the advancements made in this study, several limitations should be addressed in future research. First, while our culture method proved successful for *P. hominis* isolated from pigs, its efficacy for isolates from other host species or geographical regions remains to be determined. Additionally, the specificity of our isolation and culture techniques to *P. hominis*, as opposed to other *Trichomonas* spp., needs further investigation. Future studies should focus on adapting these methods for field applications, potentially developing portable cultivation systems for on-site isolation and identification. Moreover, comparative studies across multiple *Trichomonas* spp. using these techniques would provide valuable insights into species-specific requirements and evolutionary relationships.

The zoonotic potential of *P. hominis* highlighted in this study has significant implications for public health. Recent evidence suggests that *P. hominis* infections in humans may be more common than previously thought, particularly in immunocompromised individuals and those with close animal contact [54]. To mitigate the risk of zoonotic transmission, several measures should be considered. These include improving hygiene practices in animal husbandry, implementing regular screening programs for both animals and at-risk human populations, and developing more effective treatments for *P. hominis* infections. Furthermore, educating healthcare professionals about the potential for *P. hominis* infections in humans, especially in cases of unexplained gastrointestinal symptoms, could lead to earlier detection and treatment. Future research should focus on developing rapid diagnostic tools for field use and exploring potential vaccine candidates to prevent *P. hominis* infections in both animals and humans.

## Conclusions

In this study, a trichomonad strain obtained from fecal samples of a diarrheic pig was successfully isolated and purified employing a single-cell cloning and antibiotic selection approach. Through morphological observations and PCR with subsequent sequence analysis, the isolate was identified as *P. hominis*. Furthermore, a monoclonal sterile culture of this strain was established, which exhibited stability during in vitro transmission. These findings provide a valuable foundation for future research on the pathogenesis and treatment of trichomonad infections in pigs. The monoclonal sterile culture of *P. hominis* established through this study can serve as a reference strain for further investigations. In summary, this study underscores the significance of a comprehensive approach to isolating and characterizing trichomonad strains, thus enhancing our understanding of these pathogens and facilitating the development of effective control strategies.

## Supplementary Information

Additional file 1.

## Data Availability

No datasets were generated or analyzed during the current study.

## Abbreviations

TEM: Transmission electron microscopy
SEM: Scanning electron microscopy
FBS: Fetal bovine serum
PCR: Polymerase chain reaction
AF: Anterior flagella
RF: Recurrent flagellum
UM: Undulating membrane
P: Pelta
N: Nucleus
H: Hydrogenosomes
V: Food vacuoles
RER: Rough endoplasmic reticulum
GC: Golgi complex
SC: Striated costa
