# Supplementary material for: Development of a single-cell cloning technique for isolation of Pentatrichomonas hominis: a promising tool for diagnosing Trichomonas spp. infections in the pig breeding industry
Source: Parasit Vectors. 2025 Apr 5;18:133. doi: 10.1186/s13071-025-06752-9 (PMC11971829; doi:10.1186/s13071-025-06752-9)
Supplement: Supplementary file 1 — Additional file 1. [file 13071_2025_6752_MOESM1_ESM.docx]

**Supplementary files:**

**Development of a Single-Cell Cloning Technique for Isolation of *Pentatrichomonas hominis*: A Promising Tool for Diagnosing *Trichomonas* spp. Infections in Pig Breeding Industry**

Yibin Zhu ^1#^, Haiming Cai ^1#^, Shenquan Liao ^1^, Juan Li ^1^, Siyun Fang ^2^, Hanqin Shen ^3^, Dingai Wang ^2^, Zhuanqiang Yan ^2^, Minna Lv ^1^, Xuhui Lin ^1^, Junjing Hu ^1^, Yongle Song ^1^, Xiangjie Chen ^1^, Lijun Yin ^1^, Jianfei Zhang ^1^, Nanshan Qi ^1*^, Mingfei Sun ^1*^

^1^ Key Laboratory of Livestock Disease Prevention of Guangdong Province, Key Laboratory of Avian Influenza and Other Major Poultry Diseases Prevention and Control, Ministry of Agriculture and Rural Affairs, Institute of Animal Health, Guangdong Academy of Agricultural Sciences, Guangzhou, 510640, China.

^2^ Wen's Group Academy, Wen's Foodstuffs Group Co., Ltd., Xinxing, Guangdong 527400, China.

^3^ Guangdong Jingjie Inspection and Testing Co., Ltd., Xinxing, Guangdong 527400, China.

# These authors contributed equally.

*Corresponding author: Prof. Mingfei Sun; Dr. Nanshan Qi

E-mail address: [smf7810@126.com](mailto:smf7810@126.com), (Prof. Mingfei Sun); nanshanqi@163.com, (Dr. Nanshan Qi).

**Table S1. List of** **susceptibility test discs used at the study**

| **Antibiotics** | **Class of antibiotics** | **SMILES** ^a^ | **Product number** ^b^ | **Concentration** ^b^ |
| --- | --- | --- | --- | --- |
| Penicillin G | Penicillins | CC1([C@@H](N2[C@H](S1)[C@@H](C2=O)NC(=O)CC3=CC=CC=C3)C(=O)O)C | S1001 | 10 units/disk |
| Chloramphenicol | Chloromycetines | C1=CC(=CC=C1[C@H]([C@@H](CO)NC(=O)C(Cl)Cl)O)[N+](=O)[O-] | S1063 | 30 μg/disk |
| Erythromycin | Macrolides | CC[C@@H]1[C@@]([C@@H]([C@H](C(=O)[C@@H](C[C@@]([C@@H]([C@H]([C@@H]([C@H](C(=O)O1)C)O[C@H]2C[C@@]([C@H]([C@@H](O2)C)O)(C)OC)C)O[C@H]3[C@@H]([C@H](C[C@H](O3)C)N(C)C)O)(C)O)C)C)O)(C)O | S1039 | 15 μg/disk |
| Metronidazole | 5-nitroimidazole | CC1=NC=C(N1CCO)[N+](=O)[O-] | S1068 | 5 μg/disk |
| Amoxicillin | Penicillins | CC1([C@@H](N2[C@H](S1)[C@@H](C2=O)NC(=O)[C@@H](C3=CC=C(C=C3)O)N)C(=O)O)C | S1061 | 20 μg/disk |
| Ampicillin | Penicillins | CC1([C@@H](N2[C@H](S1)[C@@H](C2=O)NC(=O)[C@@H](C3=CC=CC=C3)N)C(=O)O)C | S1003 | 10 μg/disk |
| Ciprofloxacin | Fluoroquinolones | C1CC1N2C=C(C(=O)C3=CC(=C(C=C32)N4CCNCC4)F)C(=O)O | S1050 | 5 μg/disk |
| Enrofloxacin | Fluoroquinolones | CCN1CCN(CC1)C2=C(C=C3C(=C2)N(C=C(C3=O)C(=O)O)C4CC4)F | S1081 | 10 μg/disk |
| Lincomycin | Lincosamides | CCC[C@@H]1C[C@H](N(C1)C)C(=O)N[C@@H]([C@@H]2[C@@H]([C@@H]([C@H]([C@H](O2)SC)O)O)O)[C@@H](C)O | S1053 | 2 μg/disk |
| Amphotericin B | Polyenes | C[C@H]1/C=C/C=C/C=C/C=C/C=C/C=C/C=C/[C@@H](C[C@H]2[C@@H]([C@H](C[C@](O2)(C[C@H](C[C@H]([C@@H](CC[C@H](C[C@H](CC(=O)O[C@H]([C@@H]([C@@H]1O)C)C)O)O)O)O)O)O)O)C(=O)O)O[C@H]3[C@H]([C@H]([C@@H]([C@H](O3)C)O)N)O | S1070 | 30 μg/disk |
| Florfenicol | Amphenicols | CS(=O)(=O)C1=CC=C(C=C1)[C@H]([C@@H](CF)NC(=O)C(Cl)Cl)O | S1102 | 30 μg/disk |
| Cefoxitin | Cephalosporins | CO[C@@]1([C@@H]2N(C1=O)C(=C(CS2)COC(=O)N)C(=O)O)NC(=O)CC3=CC=CS3 | S1022 | 30 μg/disk |
| Ceftriaxone | Cephalosporins | CN1C(=NC(=O)C(=O)N1)SCC2=C(N3[C@@H]([C@@H](C3=O)NC(=O)/C(=N\OC)/C4=CSC(=N4)N)SC2)C(=O)O | S1020 | 30 μg/disk |
| Cefmetazole | Cephalosporins | CN1C(=NN=N1)SCC2=C(N3[C@@H]([C@@](C3=O)(NC(=O)CSCC#N)OC)SC2)C(=O)O | S1017 | 30 μg/disk |
| Cephalexin | Cephalosporins | CC1=C(N2[C@@H]([C@@H](C2=O)NC(=O)[C@@H](C3=CC=CC=C3)N)SC1)C(=O)O | S1011 | 30 μg/disk |
| Cefamandole | Cephalosporins | CN1C(=NN=N1)SCC2=C(N3[C@@H]([C@@H](C3=O)NC(=O)[C@@H](C4=CC=CC=C4)O)SC2)C(=O)O | S1014 | 30 μg/disk |

^a^ The SMILES (Simplified Molecular Input Line Entry System) strings provide a standardized text-based representation of the chemical structure for each antibiotic. These SMILES notations are sourced from PubChem.

^b^ Susceptibility test discs were obtained from Hangzhou Microbial Reagent Co. Ltd. (Hangzhou, China; http://www.hangwei-media.com/product/yaoming/ym1/)
